# Supplementary material for: Metagenomic Assessment of DNA Viral Diversity in Freshwater Sponges, Baikalospongia bacillifera
Source: Microorganisms. 2022 Feb 21;10(2):480. doi: 10.3390/microorganisms10020480 (PMC8876492; doi:10.3390/microorganisms10020480)
Supplement: Supplementary file 1 [file microorganisms-10-00480-s001.zip › Supplementary File S1_Materials&Methods, Tables S1-S5, Figures S1-S2.pdf]

## Supplementary Material

### Materials and Methods

*Sampling and extraction of viral DNA*

The *B. bacillifera* sponges were sampled in sterile tubes in the southern basin of Lake Baikal, near Bolshiye Koty (51°54′07.5″N, 105°06′12.0″E), at depths of about 16 m in May 2018 by divers using lightweight diving equipment as described in [1]. The two specimens of *B. bacillifera* of 5-7 $cm^3$ in volume were collected and used in this study: one looked healthy (Sv2478.2h), and another had necrosis lesions (Sv2475.1d). The sponge samples were twice washed by sterile Baikal water and thoroughly homogenized using the blender. The homogenates were frozen in nitrogen and transported to the laboratory. Then the samples were gently thawed, twice diluted with SM buffer, shaken (10,000 rpm, 30 min) with a Heidolph Multi Reax Vortex Mixer (Heidolph Instruments, Schwabach, Germany), and centrifuged at 400 g for 15 min followed by 16,000 g for 30 min. Such centrifugation mode allowed us to get rid of large and small fragments of sponges and cells, as well as to clarify and reduce the viscosity of suspensions in the best possible way. However, according to [2], the large viruses, such as *Phycodnaviridae*, *Mimiviridae*, *Poxviridae* or other nucleocytoplasmic large DNA viruses, (NCLDV) could be partially lost and further underestimated. The aqueous fraction was passed through a syringe filter with a pore size of 0.2 μm (Sartorius, Goettingen, Germany) and treated with DNase I (50 U/ml) and RNase A (100 mg/ml) enzymes (Thermo Fisher Scientific, Carlsbad, CA, USA) to remove contaminating nucleic acids.

At the same time, the control near-bottom water samples were also taken from the sponge sampling site at depths of 10, 12 and 15 m as described in [3]. The sampling was carried out by a diver using a bathometer. The water samples were filtered through 0.2-mm nitrocellulose filters (Sartorius) and combined (sample Lbw.4g). The filtrate containing virus-like particles was concentrated with a tangential flow filtration system (Sartocon Slice Ultrafiltration Set; Sartorius, Goettingen, Germany) and the Vivaspin-20 ultrafiltration device (30 kDa; Sartorius, Goettingen, Germany) to a volume of 1 ml and treated with DNase I and RNase A (Thermo Fisher Scientific, Carlsbad, CA, USA) as described above. Viral DNA was extracted from the samples of sponges and water by ZR Viral DNA kit (Zymo Research, Irvine, CA, USA) according to the manufacturer's manual. The concentration and quality of the extracted DNA were measured with a NanoDrop spectrophotometer (Thermo Fisher Scientific, Carlsbad, CA, USA) and Qubit Fluorometer (Invitrogen, Waltham, Massachusetts, USA).

*Library preparation and sequencing*

The preparation and sequencing of DNA libraries were performed in The Center of Shared Scientific Equipment "Persistence of microorganisms" of Institute for Cellular and Intracellular Symbiosis, Ural Branch of the Russian Academy of Sciences, Orenburg, Russia. The paired-end libraries were prepared using a NEBNext Ultra II FS DNA Library Prep Kit for Illumina (New England Biolabs, Ipswich, MA, USA) according to the manufacturer's protocol. The validation of DNA libraries was verified by Agilent 2100 Bioanalyzer (Agilent Technologies, Santa Clara, CA, USA). Sequencing of the libraries was conducted on the MiSeq platform (Illumina, San Diego, CA, USA) using MiSeq Reagent Kit v3 (2 x 300cycles).

Unprocessed virome reads for samples Sv2475.1d, Sv2478.2h and Lbw.4g were submitted to the National Center for Biotechnology Information (NCBI), Sequence Read Archive (SRA) database (BioProject PRJNA577390, BioSamples SAMN13025046, SAMN13025227, and SAMN16330433) [1], [3]. The direct URL to the data is as follows: https://www.ncbi.nlm.nih.gov/sra/PRJNA577390.

*Initial shotgun metagenomic data on DNA viruses in marine sponges and water samples*

For comparative analysis, we also used the datasets on marine sponge *Ianthella basta* and ocean water viromes (Great Barrier Reef (GBR), Davies Reef, sampled in January 2014; [4]) sequenced using the same library preparation and sequencing techniques as in our study (the Illumina MiSeq platform) (Table 1, the main text). SRA archives of

these data sets (raw reads in FASTQ files) were downloaded from the NCBI database using the "fastq-dump" utility.

The paired reads of marine viromes were combined into one FASTQ dataset together with the Baikal ones; then joint primary processing of paired reads was carried out as described below. All data were used for a hybrid metagenomic assembly (cross-assembly) in one round of data analysis. Replicates of marine viral metagenomes from similar samples were combined before future analysis.

*Primary processing of virome reads*

The quality visualization of the virome datasets (paired reads) was carried out using the FASTQC program (http://www.bioinformatics.babraham.ac.uk/projects/fastqc; accessed on 8 January 2019). Trimming of reads by the quality was carried out with the Trimmomatic V 0.39 program [5] using the adaptive quality trimmer options (MAXINFO: 40: 0.1); the reads of 100 bp or more were used for further analysis.

To determine the proportions of viral and non-viral reads in the datasets (DNA of viruses, eukaryotes, prokaryotes, archaea and unclassified DNA), all reads were compared with the amino acid sequences of the UNIPROT database (UniRef50) [6] using the BLASTx algorithm [7] with the following parameters: word size for word finder algorithm, 6; cost to open a gap, 6; gap extension cost, 2; e-value ≤ 0.00001; bit score ≥ 50, and identity ≥ 35%. The search was carried out with the standard genetic code and the genetic code of bacteria and archaea. For each read matching the UniRef50 database, a high-ranking taxon (Virus, Eukaryote, Bacterium, or Archaea) from the UNIPROT description was assigned.

*Assembly of virome reads; identification and taxonomic assignment of viral scaffolds*

The assembly of viral reads and further taxonomic identification of viral scaffolds was carried out as reported before in the study of water samples from different areas of Lake Baikal [8]. Briefly, the SPAdes 3.13.1 metagenomics assembler, metaSPAdes [9], with parameters of paired-end reads and K-mer lengths of 21, 33, 55, and 77 were used for the de novo cross-assembly of data sets. The scaffolds with coverage more than 5 and a length of ≥5000 bp were used for further analysis.

The VirSorter tool [10] on the «CYVERSE» Discovery Environment web server (https://de.cyverse.org/de/; accessed on 19 July 2020) was used for identification of the viral scaffolds and open reading frames (ORFs) in them.

Taxonomic identification for the viral scaffolds was carried out by comparisons of predicted viral proteins in scaffolds with the NCBI RefSeq complete viral proteome database and comparisons of viral scaffold with NCBI RefSeq complete viral genome database [11].

The proteome comparison of proteins was carried out by the BLASTp algorithm [7] with the following parameters: word size, 6; gap open cost, 6; gap extension cost, 2; e-value ≤ 0.00001; bit score ≥ 50, and identity ≥ 35%. For each protein in the scaffold, the best match in terms of the bit score value was selected. If a single scaffold had multiple proteins that matched different taxa (NCBI RefSeq ID), the one with the largest number of matching proteins was chosen as the most closely related virus taxon (virotype) of this scaffold. If the proteins were not repeated in the match list, the level of similarity of the matched proteins was taken into account, and the NCBI RefSeq taxon (ID) with the highest percentage of protein similarity was selected as the virotype.

If no match with the viral proteome was found for the scaffold, then its nucleotide sequence was matched with the nucleotide sequences of viral genomes from the NCBI RefSeq by BLASTn algorithm [7] having the following parameters: cost to open a gap, 2; gap extension cost, 1; word size, 9; penalty for a nucleotide mismatch, 1; reward, 1; e-value of ≤ 0.00001, bit score ≥ 50. For each metaviromic assembly scaffold, for which a match with NCBI RefSeq was found, the proportion of the total scaffold length covered by alignment with the reference viral genome was determined. A virus taxon from NCBI RefSeq with the highest proportion of coverage in alignments of the nucleotide sequences was chosen as the scaffold virotype identifier.

The advantage of our approach is the combined analysis of complete viral genomes and proteomes that allowed us to compare the reads with viral genomes, for which the annotation of their proteome was not

represented in the NCBI RefSeq database, and, at the same time, to identify the distant similarity of the reads, comparing translated reads with proteins.

The Burrows-Wheeler Aligner (BWA) software [12] was used to map paired-end reads on scaffolds and calculate the total coverage of viral scaffolds in the assembly and coverage of scaffolds by reads from each sample. The BWA results were used to determine the number of reads mapped on each predicted viral scaffold from each sample. Counts of the predicted viral proteins (ORFs) in samples were defined as the number of reads mapped on a scaffold containing a given protein. Consequently, the count table of viral scaffold representation in the analyzed samples was constructed.

The count table of scaffolds (number of hits per each virotype in the sample) was normalized to the scaffolds length according to the algorithm from our previous study [8].

*Statistical analysis of taxonomic diversity*

The potential (underestimated) number of virus scaffolds and virotypes (species richness) in communities was evaluated using Chao1 [13] and ACE [14] indices. Shannon and Simpson indices [15] of biodiversity were also calculated (Table 2, the main text) for virus scaffolds and virotypes.

For multivariate statistical analyses, the taxonomic composition based on the scaffolds count table (including scaffolds not identified before the virotype) was normalized to the relative abundance of reads per sample. To equalize the effect of scaffolds with different counts per sample (from the highest to the lowest ones), the values ranged between 0 and 1.

The taxonomic composition similarity of the samples (similarity in virus scaffold count table per samples) was visualized using hierarchical cluster analysis by the “average” method with bootstrap support calculation of clustering in the “pvclust” [16] package for the R programming language and the nonmetric multidimensional scaling (NMDS) ordination method with the Bray–Curtis dissimilarity metric. Gradient vectors of the viral family composition were fitted on the NMDS scatter plot. The reliability of linear approximation for gradient vectors was assessed by multivariate linear regression analysis.

Biodiversity analysis and NMDS were carried out in the “vegan” package for the R programming language [17] according to the tutorials [18].

Dominant scaffolds and virotypes in Baikal and marine samples were visualized with the heat map generated using the “gplots” [19] package in R. Columns (samples) in the heat map were clustered and grouped in similarity order (i.e., Bray–Curtis distance metric and the complete-link clustering method).

The significance of the difference between the samples in counts of virotype reads was assessed using the chi-square test for independence. The p-value for the chi-square test was adjusted by the Bonferroni correction for multiple hypothesis testing.

*Functional assignment of viral communities*

Functional assignment of predicted viral proteins (ORFs) was carried out in three different ways.

First way. Viral proteins (ORFs) were matched with the UniProtKB/Swiss-Prot database [6] by the BLASTp algorithm with the following parameters: word size for word finder algorithm, 6; cost to open a gap, 6; gap extension cost, 2. Viral proteins were considered identified if the best hits had e-value ≤ 0.00001, bit score ≥ 50 and identity ≥ 35%. To describe the predicted functions of proteins, the KEGG (Kyoto Encyclopedia of Genes and Genomes) Orthology (KO) identifiers [20] were taken from annotations uploaded from UniProtKB/Swiss-Prot.

Second way. Viral proteins (ORFs) were matched with functional motifs of proteins in the Pfam database [21] using an online resource (https://www.ebi.ac.uk/Tools/pfa/pfamscan/; accessed on 20 September 2021) [22]. All detected Pfam ID of Pfam motifs among viral proteins were processed according to the following algorithm: among all protein annotations uploaded from UniProtKB/Swiss-Prot, the Pfam ID advisers of viral proteins were found; the corresponding KEGG Orthology (KO) identifiers were found in these UniProt annotations; these founded KEGG Orthology (KO) identifiers were used to describe the functions of the viral proteins.

Third way. Viral proteins (ORFs) were matched with functional motifs of proteins in the KOfam database using an online resource (https://www.genome.jp/tools/kofamkoala/; accessed on 20 September 2021) [23]. This analysis provided a direct description of viral proteins in KEGG Orthology (KO) identifiers.

To describe each viral protein, all possible KO identifiers obtained in three types of analysis were used because the same protein can have several alternative functions. The use of three alternative databases in functional analysis has significantly expanded the list of viral proteins with the functional assignment.

The KO identifiers of viral proteins were processed in the «KEGGREST» package [24] for R programming language to obtain the KEGG pathway classification (https://www.genome.jp/kegg/pathway.html; accessed on 4 July 2020). The count of the predicted viral proteins in samples was transformed into counts of the KEGG pathway classification groups that were normalized for the average number of hits on the viral proteins in each sample.

KEGG pathway classification allowed us to detect auxiliary metabolic genes (AMGs) among predicted viral proteins as a group belonging to the global metabolic category. The counts of AMGs viral proteins in different samples were visualized with a heat map generated using the «gplots» package [19] in R. Columns (samples) in the heat map were clustered and grouped in similarity order (i.e., Bray–Curtis distance metric and the complete-link clustering method).

*Viral hosts prediction*

Host prediction for the set of viral scaffolds was carried out by the method described previously [8]. Briefly, the method was based on the Virus–Host database [25]. After taxonomic identification of predicted viral scaffolds, the list of corresponding hosts from the Virus–Host database was obtained. The count of the predicted viral scaffolds was transformed into tables representing DNA viruses (virotypes) that infect a certain host species. This table was used to construct the representation gradients vectors of host taxa in the NMDS scatter plot of viral scaffolds count table comparisons.

*Bacterial defense mechanisms against viruses*

The genomic assemblies of two bacterial strains, *Flavobacterium* sp. Strain SLB02 and *Janthinobacterium* sp. Strain SLB01, isolated from the diseased Baikal sponge *Lubomirskia baikalensis* were recently published [26]. In our study, we analyzed in these strains the presence of any defense mechanisms against the viruses that we revealed in the Baikal sponge *B. bacillifera.* The defense systems in the genomes of these bacteria we were detected using the Prokaryotic Antiviral Defense LOCator (PADLOC) online service (https://padloc.otago.ac.nz/padloc/ accessed on February 5, 2022) [27].

Bacterial genomes were also searched for the CRISPR-Cas systems using the CRISPRCasFinder online service (https://crisprcas.i2bc.paris-saclay.fr/CrisprCasFinder/Index accessed on February 5, 2022) [28]. The CRISPRCas spacers were compared with viral scaffolds using the blastn-short algorithm [7] as recommended in [29] (a maximum expect value of 1; a gap opening penalty 10; a gap extension penalty 2; a mismatch penalty 1; a word size 7; and dust filtering turned off).

## Supplementary Tables

**Table S1.** The percentage of viral families in samples of marine and freshwater sponges, and in water samples.

| Family | Known hosts | GBR.sw | I.basta.d | I.basta.h | I.basta.md | I.basta.nd | LBw.4g | Sv2475.1d | Sv2478.2h |
|---|---|---|---|---|---|---|---|---|---|
| *Myoviridae* | bacteria | 11.67 | 40.57 | 32.39 | 43.80 | 46.16 | 12.11 | 11.30 | 6.61 |
| *Podoviridae* | bacteria | 24.98 | 26.47 | 20.37 | 25.33 | 21.56 | 20.20 | 30.62 | 29.87 |
| *Siphoviridae* | bacteria | 29.62 | 2.69 | 3.14 | 1.95 | 1.81 | 45.18 | 35.78 | 38.56 |
| unknown | - | 18.80 | 12.95 | 32.63 | 13.00 | 12.88 | 19.00 | 14.45 | 17.17 |
| *Poxviridae* | arthropoda, vertebrates | 0.18 | 8.01 | 5.37 | 7.37 | 8.43 | 0.01 | 0.02 | 0.06 |
| *Phycodnaviridae* | algae | 7.16 | 4.03 | 2.74 | 4.12 | 4.17 | 0.20 | 0.34 | 0.68 |
| *Lavidaviridae* | protozoa/viruses | 0.00 | 0.00 | 0.00 | 0.00 | 0.00 | 3.11 | 6.27 | 5.24 |
| *Ackermannviridae* | bacteria | 0.01 | 3.82 | 2.52 | 3.06 | 3.54 | 0.01 | 0.01 | 0.04 |
| *Microviridae* | bacteria | 6.36 | 0.14 | 0.00 | 0.32 | 0.38 | 0.00 | 0.00 | 0.00 |
| *Mimiviridae* | protozoa | 0.00 | 1.29 | 0.81 | 1.05 | 1.02 | 0.02 | 0.17 | 0.32 |
| unclassified | bacteria and others | 1.22 | 0.00 | 0.00 | 0.00 | 0.00 | 0.08 | 0.16 | 0.27 |
| *Baculoviridae* | arthropods, crustaceans | 0.00 | 0.00 | 0.00 | 0.00 | 0.00 | 0.04 | 0.36 | 0.44 |
| *Bicaudaviridae* | archaea | 0.00 | 0.00 | 0.00 | 0.00 | 0.00 | 0.02 | 0.29 | 0.42 |
| *Herelleviridae* | bacteria | 0.00 | 0.04 | 0.04 | 0.00 | 0.04 | 0.02 | 0.21 | 0.32 |

**Table S2.** Viral scaffolds mostly represented in the Baikal samples by the number of reads and closely related viruses (virotypes); the maximum and average similarity (in %) of predicted viral proteins with the NCBI RefSeq database (the ten largest sets of reads corresponding to specific scaffolds and virotype in each sample are marked in bold).

| Scaffolds | RefSeq_ID | Coverage | Average Similarity | Max Similarity | Virotype | Viral family | LBw.4g | Sv2475.1d | Sv2478.2h |
|---|---|---|---|---|---|---|---|---|---|
| NODE_3188_length_5340 | 567 | 8.03 | 36.70 | 36.70 | *Arthrobacter phage Decurro* | *Siphoviridae* | **5.01** | **3.45** | **2.18** |
| NODE_2173_length_6490 | 296 | 3.98 | 46.00 | 46.00 | *Enterobacteria phage Sf101* | *Podoviridae* | 0.00 | **9.00** | 0.79 |
| NODE_1230_length_8743 | 2979 | 13.83 | 36.50 | 36.50 | *Bdellovibrio phage phi1422* | *Myoviridae* | 0.00 | **7.09** | 0.79 |
| NODE_1398_length_8224 | 3489 | 19.95 | 43.20 | 43.20 | *Cellulophaga phage phi10:1* | *Siphoviridae* | 0.26 | **3.60** | **3.86** |
| NODE_1239_length_8715 | 3483 | 46.75 | 35.70 | 35.70 | *Cellulophaga phage phi38:1* | *Podoviridae* | 0.23 | **2.97** | **3.71** |
| NODE_2108_length_6569 | 3483 | 66.04 | 36.90 | 38.40 | *Cellulophaga phage phi38:1* | *Podoviridae* | 0.24 | **2.92** | **3.71** |
| NODE_3149_length_5378 | 0 | 0.00 | 0.00 | 0.00 | unknown | unknown | **2.41** | **1.62** | 1.10 |
| NODE_1440_length_8088 | 2705 | 8.72 | 47.45 | 58.80 | *Synechococcus phage ACG-2014h* | *Myoviridae* | **4.04** | 0.35 | 0.35 |
| NODE_36_length_45225 | 2687 | 4.67 | 51.07 | 63.50 | *Croceibacter phage P2559Y* | *Siphoviridae* | 0.00 | **4.24** | 0.45 |
| NODE_921_length_10196 | 0 | 0.00 | 0.00 | 0.00 | unknown | unknown | 0.14 | **2.19** | **2.21** |
| NODE_1603_length_7601 | 5925 | 5.29 | 35.00 | 35.00 | *Synechococcus phage S-SKS1* | *Myoviridae* | **2.90** | 0.29 | 0.27 |
| NODE_113_length_33335 | 4179 | 5.41 | 60.69 | 64.46 | *Cellulophaga phage phi19:3* | *Podoviridae* | 0.10 | 1.43 | **1.92** |
| NODE_2563_length_5998 | 5895 | 6.30 | 45.20 | 45.20 | *Prochlorococcus phage P-GSP1* | *Podoviridae* | **2.88** | 0.25 | 0.26 |
| NODE_1962_length_6848 | 3483 | 30.88 | 37.85 | 38.60 | *Cellulophaga phage phi38:1* | *Podoviridae* | 0.14 | 1.33 | **1.74** |
| NODE_3368_length_5179 | 8987 | 15.06 | 40.80 | 40.80 | *Xylella phage Sano* | *Siphoviridae* | **2.54** | 0.32 | 0.33 |
| NODE_192_length_26621 | 520 | 3.69 | 36.85 | 37.90 | *Yellowstone Lake virophage 5* | *Lavidaviridae* | 0.39 | **1.58** | 1.07 |
| NODE_31_length_45884 | 3483 | 9.77 | 35.65 | 36.30 | *Cellulophaga phage phi38:1* | *Podoviridae* | 0.10 | 1.23 | **1.57** |
| NODE_63_length_38877 | 3483 | 9.44 | 37.30 | 37.40 | *Cellulophaga phage phi38:1* | *Podoviridae* | 0.20 | 1.05 | **1.56** |
| NODE_2892_length_5625 | 567 | 7.04 | 44.80 | 44.80 | *Arthrobacter phage Decurro* | *Siphoviridae* | **1.71** | 0.60 | 0.42 |
| NODE_181_length_27777 | 3483 | 5.51 | 36.30 | 36.30 | *Cellulophaga phage phi38:1* | *Podoviridae* | 0.07 | 1.02 | **1.60** |
| NODE_2010_length_6733 | 0 | 0.00 | 0.00 | 0.00 | unknown | unknown | **2.10** | 0.27 | 0.27 |
| NODE_2403_length_6206 | 0 | 0.00 | 0.00 | 0.00 | unknown | unknown | **2.14** | 0.06 | 0.06 |
| NODE_1418_length_8150 | 419 | 6.40 | 37.20 | 37.20 | Staphylococcus phage SA1 | *Myoviridae* | **1.50** | 0.24 | 0.19 |

**Table S3.** The percentage of putative host taxa predicted for viruses in samples.

| Host taxonomy (Phylum or family) | GBR.sw | I.basta.h | I.basta.d | I.basta.md | I.basta.nd | LBw.4g | Sv2475.1d | Sv2478.2h |
|---|---|---|---|---|---|---|---|---|
| Bacteria_Bacteroidetes | 15.78 | 24.37 | 26.57 | 24.81 | 21.68 | 3.06 | 32.39 | 38.83 |
| Bacteria_Proteobacteria | 43.99 | 13.20 | 14.86 | 15.26 | 15.77 | 21.50 | 20.62 | 8.97 |
| Bacteria_Cyanobacteria | 3.53 | 22.83 | 26.80 | 29.87 | 30.09 | 26.27 | 6.04 | 7.91 |
| Bacteria_Actinobacteria | 2.96 | 0.06 | 0.05 | 0.03 | 0.02 | 24.17 | 15.26 | 16.84 |
| Bacteria_Firmicutes | 4.10 | 0.02 | 0.02 | 0.00 | 0.03 | 4.25 | 1.26 | 1.66 |
| Bacteria_Chlamydiae | 0.81 | 0.00 | 0.00 | 0.00 | 0.00 | 0.00 | 0.00 | 0.00 |
| Bacteria_Verrucomicrobia | 0.46 | 0.00 | 0.00 | 0.00 | 0.00 | 0.02 | 0.01 | 0.01 |
| Bacteria_unclassified | 0.46 | 0.00 | 0.00 | 0.00 | 0.00 | 0.02 | 0.01 | 0.01 |
| Eukaryota_Arthropoda | 0.79 | 14.87 | 20.10 | 18.44 | 21.05 | 0.05 | 0.35 | 0.53 |
| Eukaryota_Chlorophyta | 0.11 | 1.85 | 2.49 | 2.54 | 2.52 | 0.25 | 0.32 | 0.66 |
| Eukaryota_Haptophyceae | 6.28 | 0.00 | 0.00 | 0.00 | 0.00 | 0.00 | 0.00 | 0.00 |
| Eukaryota_Bicosoecida | 0.00 | 0.54 | 0.80 | 0.64 | 0.60 | 0.00 | 0.00 | 0.00 |
| Eukaryota_Amoebozoa | 0.00 | 0.03 | 0.02 | 0.03 | 0.04 | 0.04 | 0.44 | 0.79 |
| Archaea_Crenarchaeota | 0.00 | 0.00 | 0.00 | 0.00 | 0.00 | 0.03 | 0.49 | 0.69 |
| Archaea_Euryarchaeota | 0.28 | 0.00 | 0.00 | 0.00 | 0.00 | 0.39 | 0.07 | 0.06 |
| Viruses_Mimiviridae | 0.00 | 0.00 | 0.00 | 0.00 | 0.00 | 2.07 | 6.13 | 5.00 |
| Viruses_Caudovirales | 0.86 | 0.00 | 0.00 | 0.00 | 0.00 | 0.00 | 0.00 | 0.00 |
| Viruses_unclassified | 0.00 | 0.00 | 0.00 | 0.00 | 0.00 | 0.22 | 0.61 | 0.56 |
| unknown_unknown | 19.58 | 22.23 | 8.31 | 8.36 | 8.19 | 17.68 | 16.01 | 17.49 |

**Table S4.** The antiviral defense systems revealed in assembled genomes of *Janthinobacterium* sp. SLB01 and *Flavobacterium sp.* SLB02 isolated from the Baikal sponge *Lubomirskia baikalensis (*diseased).

| System | Protein name | Target name | SeqID | Start | End | Strand | Relative position |
|---|---|---|---|---|---|---|---|
| ***Janthinobacterium sp.* SLB01** | | | | | | | |
| dXTPase | dGTPase | F3B38_RS03280 | NZ_VZAB01000001.1 | 767216 | 768353 | + | 642 |
| zorya_type_I | ZorA1 | F3B38_RS04530 | NZ_VZAB01000001.1 | 1124824 | 1126888 | + | 888 |
| zorya_type_I | ZorB1 | F3B38_RS04535 | NZ_VZAB01000001.1 | 1126884 | 1127601 | + | 889 |
| zorya_type_I | ZorC1 | F3B38_RS04540 | NZ_VZAB01000001.1 | 1127660 | 1129271 | + | 890 |
| zorya_type_I | ZorD1 | F3B38_RS04545 | NZ_VZAB01000001.1 | 1129271 | 1133024 | + | 891 |
| septu_type_I | PtuB1 | F3B38_RS17795 | NZ_VZAB01000002.1 | 1490356 | 1491079 | - | 1299 |
| septu_type_I | PtuA1 | F3B38_RS17800 | NZ_VZAB01000002.1 | 1491071 | 1492544 | - | 1300 |
| gabija | GajB | F3B38_RS15530 | NZ_VZAB01000002.1 | 979745 | 981398 | - | 850 |
| gabija | GajA | F3B38_RS15535 | NZ_VZAB01000002.1 | 981406 | 983158 | - | 851 |
| ***Flavobacterium sp.* SLB02** | | | | | | | |
| dXTPase | dGTPase | GIY83_17275 | CP045928.1 | 4622626 | 4623973 | - | 3370 |
| cbass_type_III | Effector | GIY83_03730 | CP045928.1 | 859116 | 859962 | - | 720 |
| cbass_type_III | TRIP13 | GIY83_03735 | CP045928.1 | 859958 | 860843 | - | 721 |
| cbass_type_III | HORMA | GIY83_03740 | CP045928.1 | 860842 | 861355 | - | 722 |
| cbass_type_III | Cyclase | GIY83_03745 | CP045928.1 | 861357 | 862338 | - | 723 |

**Table S5.** Matches revealed between CRISPR-Cas spacers from bacterial strains of *Janthinobacterium sp.* Strain SLB01 and *Flavobacterium sp.* Strain SLB02 (isolated from the Baikal sponge *Lubomirskia baikalensis,* [26]) and viral scaffolds from the sponge *Baikalospongia bacillifera*.

| Bacterial strains | Spacer ID | Nucleotide match length | Scaffold ID | Scaffold taxon (NCBI RefSeq)* | Host taxon (Virus Host database)* |
|---|---|---|---|---|---|
| SLB01 | SLB01_1 | 10 | NODE_201_length_26043 | Viruses; Duplodnaviria; Heunggongvirae; Uroviricota; Caudoviricetes; Caudovirales; Podoviridae; **Dunaliella viridis virus SI2** | Eukaryota; Viridiplantae; Chlorophyta; Chlorophyceae; Chlamydomonadales; Dunaliellaceae; Dunaliella; **Dunaliella viridis** |
| SLB02 | SLB02_1 | 7 | NODE_201_length_26043 | Viruses; Duplodnaviria; Heunggongvirae; Uroviricota; Caudoviricetes; Caudovirales; Podoviridae; **Dunaliella viridis virus SI2** | Eukaryota; Viridiplantae; Chlorophyta; Chlorophyceae; Chlamydomonadales; Dunaliellaceae; Dunaliella; **Dunaliella viridis** |
| | SLB02_1 | 8 | NODE_2095_length_6599 | Viruses; Varidnaviria; Bamfordvirae; Nucleocytoviricota; Megaviricetes; Algavirales; Phycodnaviridae; Prasinovirus; unclassified Prasinovirus; **Yellowstone lake phycodnavirus 1** | Unknown |
| | SLB02_1 | 8 | NODE_147_length_30473 | Viruses; Duplodnaviria; Heunggongvirae; Uroviricota; Caudoviricetes; Caudovirales; Siphoviridae; **Bacteroides phage B124-14** | Bacteria; Bacteroidetes; Bacteroidia; Bacteroidales; Bacteroidaceae; Bacteroides; **Bacteroides fragilis** |
| | SLB02_3 | 14 | NODE_3198_length_5331 | Viruses; Duplodnaviria; Heunggongvirae; Uroviricota; Caudoviricetes; Caudovirales; Siphoviridae; **Bacteroides phage B40-8** | Bacteria; Proteobacteria; Alphaproteobacteria; Pelagibacterales; Pelagibacteraceae; Candidatus Pelagibacter; **Candidatus Pelagibacter ubique HTCC1062** |
| | SLB02_3 | 15 | NODE_129_length_31556 | Viruses; Duplodnaviria; Heunggongvirae; Uroviricota; Caudoviricetes; Caudovirales; Siphoviridae; **Cyanophage KBS-S-2A** | Bacteria; Cyanobacteria; Synechococcales; Synechococcaceae; Synechococcus; **Synechococcus sp. WH 7803** |
| | SLB02_4 | 10 | NODE_549_length_13787 | Viruses; Duplodnaviria; Heunggongvirae; Uroviricota; Caudoviricetes; Caudovirales; Siphoviridae; **Bacillus phage vB_BhaS-171** | Bacteria; Terrabacteria group; Firmicutes; Bacilli; Bacillales; Bacillaceae; Sutcliffiella; **Sutcliffiella halmapala** |
| | SLB02_4 | 11 | NODE_296_length_20467 | Viruses; Duplodnaviria; Heunggongvirae; Uroviricota; Caudoviricetes; Caudovirales; Siphoviridae; **Idiomarinaceae phage 1N2-2** | Bacteria; Proteobacteria; Gammaproteobacteria; Alteromonadales; Idiomarinaceae; **Idiomarinaceae bacterium N2-2** |
| | SLB02_4 | 12 | NODE_201_length_26043 | Viruses; Duplodnaviria; Heunggongvirae; Uroviricota; Caudoviricetes; Caudovirales; Podoviridae; **Dunaliella viridis virus SI2** | Eukaryota; Viridiplantae; Chlorophyta; Chlorophyceae; Chlamydomonadales; Dunaliellaceae; Dunaliella; **Dunaliella viridis** |
| | SLB02_5 | 8 | NODE_21_length_51481 | Viruses; Duplodnaviria; Heunggongvirae; Uroviricota; Caudoviricetes; Caudovirales; Myoviridae; **Cellulophaga phage phi38:1** | Bacteria; Bacteroidetes; Flavobacteriia; Flavobacteriales; Flavobacteriaceae; Cellulophaga; **Cellulophaga baltica NN016038** |
| | SLB02_5 | 10 | NODE_2619_length_5942 | Viruses; Duplodnaviria; Heunggongvirae; Uroviricota; Caudoviricetes; Caudovirales; Myoviridae; Palaemonvirus; Prochlorococcus virus PSSM7; **Prochlorococcus phage P-SSM7** | Bacteria; Terrabacteria group; Cyanobacteria/Melainabacteria group; Cyanobacteria; Synechococcales; Prochlorococcaceae; Prochlorococcus; Prochlorococcus marinus**; Prochlorococcus marinus str. NATL1A** |
| | SLB02_5 | 7 | NODE_186_length_27247 | Viruses; Duplodnaviria; Heunggongvirae; Uroviricota; Caudoviricetes; Caudovirales; Siphoviridae; Inhaviru; **Nonlabens phage P12024L** | Bacteria; Bacteroidetes; Flavobacteriia; Flavobacteriales; **Flavobacteriaceae; Nonlabens** |
| | SLB02_6 | 12 | NODE_139_length_30907 | Viruses; Duplodnaviria; Heunggongvirae; Uroviricota; Caudoviricetes; Caudovirales; Siphoviridae; Inhaviru; **Nonlabens phage P12024L** | Bacteria; Bacteroidetes; Flavobacteriia; Flavobacteriales; **Flavobacteriaceae; Nonlabens** |
| | SLB02_6 | 9 | NODE_177_length_28020 | Viruses; Duplodnaviria; Heunggongvirae; Uroviricota; Caudoviricetes; Caudovirales; Siphoviridae; **Cyanophage KBS-S-2A** | Bacteria; Cyanobacteria; Synechococcales; Synechococcaceae; Synechococcus; **Synechococcus sp. WH 7803** |

*Bold font indicates species names of virotypes hosts. Nucleotide sequences of CRISPR-Cas spacer are presented in the Supplementary File S3 (SLB01-SLB02.fasta), the result of blast-short alignment is given in the Supplementary File S4 (SLB01-SLB02.out).

## Supplementary Figures

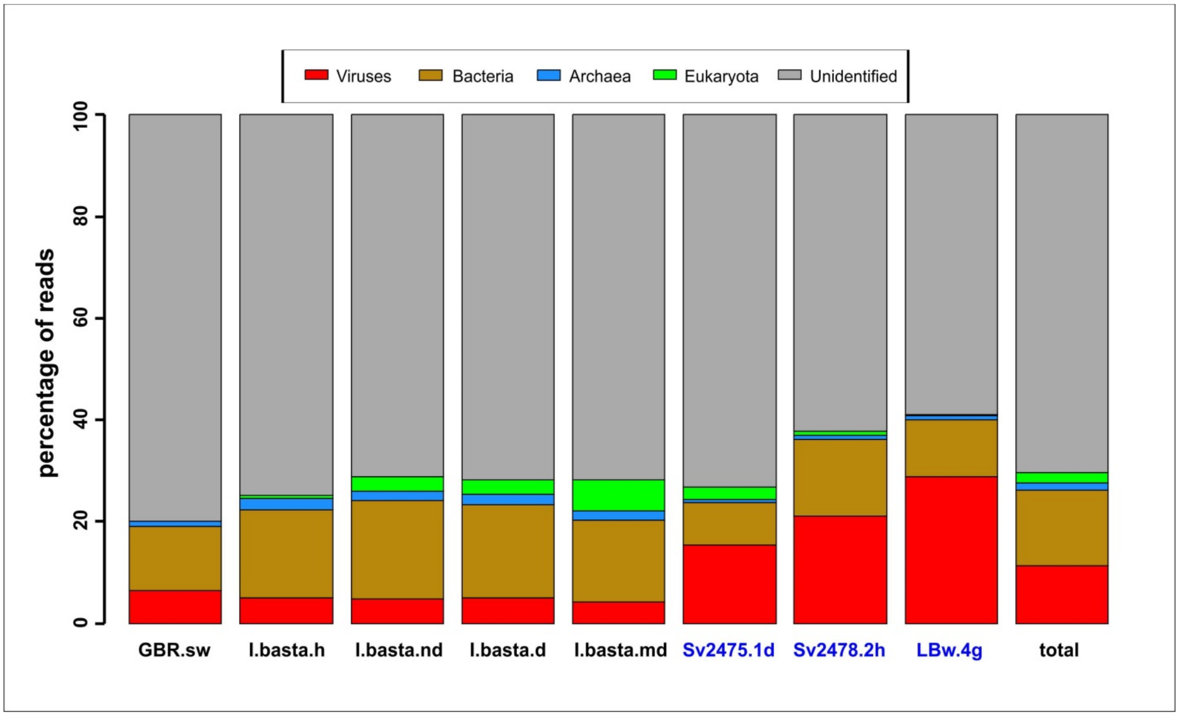


**Figure S1.** The percentage of the reads affiliated to Viruses, Bacteria, Archaea and Eukaryota.

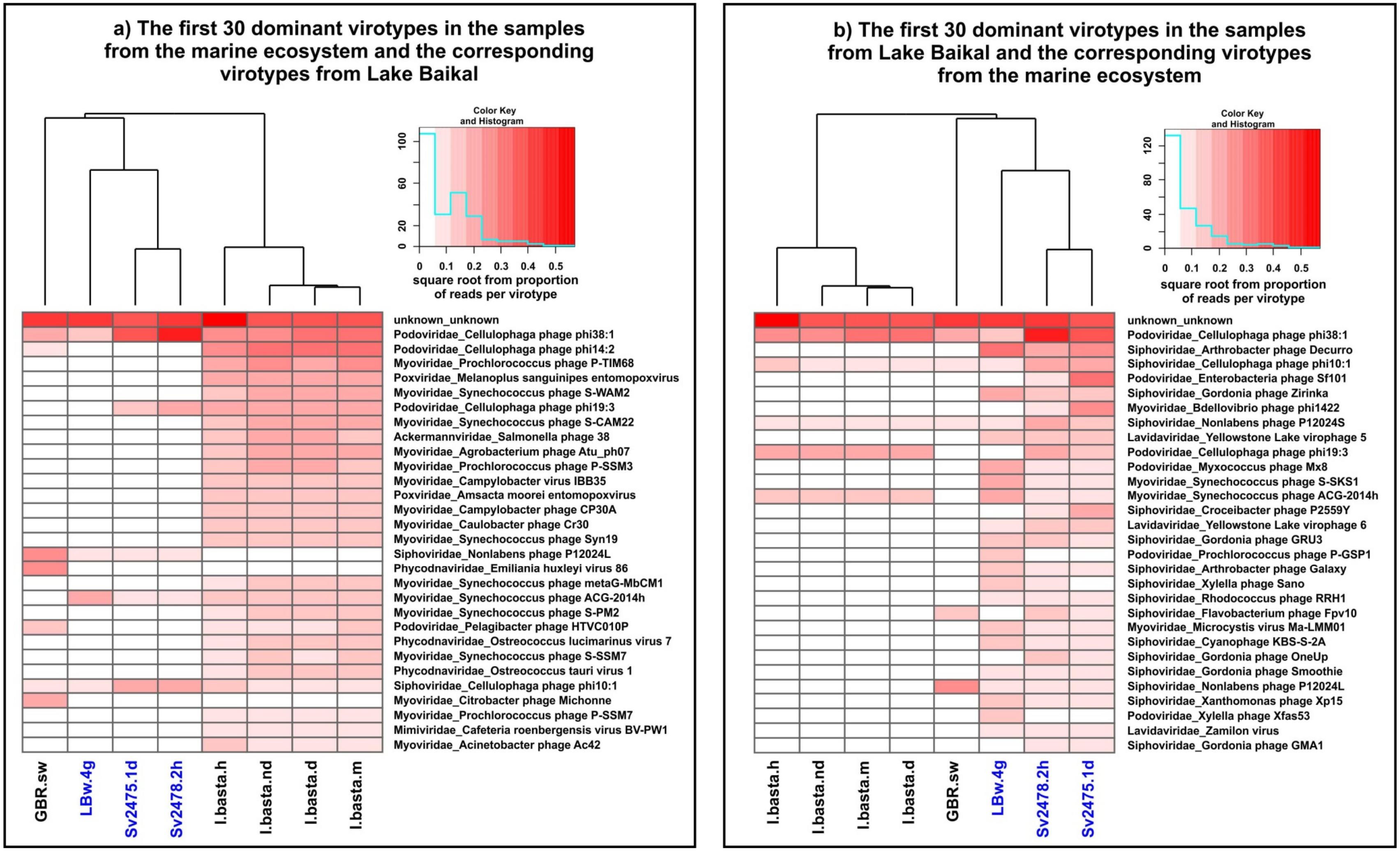


**Figure S2.** Heat maps demonstrating the number of reads for dominant virotypes in samples from marine and freshwater ecosystems: (**a**) Representation of dominant virotypes in marine vs. freshwater samples, and, conversely; (**b**) representation of dominant virotypes in freshwater vs. marine samples.

## Referenses


1. Butina, T. V.; Khanaev, I. V.; Kravtsova, L. S.; Maikova, O. O.; Bukin, Y. S. Metavirome datasets from two endemic Baikal sponges Baikalospongia bacillifera. *Data Br.* **2020**, *29*, 105260.

2. Conceição-Neto, N.; Zeller, M.; Lefrère, H.; De Bruyn, P.; Beller, L.; Deboutte, W.; Yinda, C. K.; Lavigne, R.; Maes, P.; Ranst, M. Van; et al. Modular approach to customise sample preparation procedures for viral metagenomics: A reproducible protocol for virome analysis. *Sci. Rep.* **2015**, *5*, 1–14.

3. Butina, T. V.; Petrushin, I. S.; Khanaev, I. V.; Bukin, Y. S. Virome Analysis of Near-Bottom Coastal Water of Lake Baikal. *Microbiol. Resour. Announc.* **2020**, *9*, 1–3.

4. Laffy, P. W.; Wood-Charlson, E. M.; Turaev, D.; Jutz, S.; Pascelli, C.; Botté, E. S.; Bell, S. C.; Peirce, T. E.; Weynberg, K. D.; van Oppen, M. J. H.; et al. Reef invertebrate viromics: diversity, host specificity and functional capacity. *Environ. Microbiol.* **2018**, *20*, 2125–2141.

5. Bolger, A. M.; Lohse, M.; Usadel, B. Trimmomatic: A flexible trimmer for Illumina sequence data. *Bioinformatics* **2014**, *30*, 2114–2120.

6. Bairoch, A.; Apweiler, R.; Wu, C. H.; Barker, W. C.; Boeckmann, B.; Ferro, S.; Gasteiger, E.; Huang, H.; Lopez, R.; Magrane, M.; et al. The Universal Protein Resource (UniProt). *Nucleic Acids Res.* **2005**, *33*, D154–D159.

7. Altschul, S. F.; Gish, W.; Miller, W.; Myers, E. W.; Lipman, D. J. Basic local alignment search tool. *J. Mol. Biol.* **1990**, *215*, 403–410.

8. Butina, T. V.; Bukin, Y. S.; Petrushin, I. S.; Tupikin, A. E.; Kabilov, M. R.; Belikov, S. I. Extended evaluation of viral diversity in lake baikal through metagenomics. *Microorganisms* **2021**, *9*, 1–31.

9. Nurk, S.; Meleshko, D.; Korobeynikov, A.; Pevzner, P. A. MetaSPAdes: A new versatile metagenomic assembler. *Genome Res.* **2017**, *27*, 824–834.

10. Roux, S.; Enault, F.; Hurwitz, B. L.; Sullivan, M. B. VirSorter: Mining viral signal from microbial genomic data. *PeerJ* **2015**, *3*, e985.

11. Pruitt, K. D.; Tatusova, T.; Maglott, D. R. NCBI Reference Sequence (RefSeq): A curated non-redundant sequence database of genomes, transcripts and proteins. *Nucleic Acids Res.* **2005**, *33*, D501–D504.

12. Li, H.; Durbin, R. Fast and accurate short read alignment with Burrows-Wheeler transform. *Bioinformatics* **2009**, *25*, 1754–1760.

13. O'Hara, R. B. Species richness estimators: How many species can dance on the head of a pin? *J. Anim. Ecol.* **2005**, *74*, 375–386.

14. Colwell, R. K.; Coddington, J. A. Estimating terrestrial biodiversity through extrapolation. *Biodivers. Meas. Estim.* **1995**, *345*, 101–118.

15. Hill, M. O. Diversity and Evenness: A Unifying Notation and Its Consequences. *Ecology* **1973**, *54*, 427–432.

16. Suzuki, R.; Shimodaira, H. Pvclust: An R package for assessing the uncertainty in hierarchical clustering. *Bioinformatics* **2006**, *22*, 1540–1542.

17. Oksanen, J.; Kindt, R.; Legendre, P.; O'Hara, B.; Simpson, G. L.; Solymos, P. M.; Stevens, M. H. H.; & Wagner, H. The vegan package. *Community Ecol. Packag.* **2008**, 190.

18. Oksanen, J. Vegan: ecological diversity. *R Proj.* **2018**.

19. Warnes, G. R.; Bolker, B.; Bonebakker, L.; Gentleman, R.; Liaw, W. H. A.; Lumley, T.; Maechler, M.; Magnusson, A.; Moeller, S.; Schwartz, M.; et al. Package "gplots": Various R programming tools for plotting data. *R Packag. version 2.17.0.* **2016**, 1–68.

20. Mao, X.; Cai, T.; Olyarchuk, J. G.; Wei, L. Automated genome annotation and pathway identification using the KEGG Orthology (KO) as a controlled vocabulary. *Bioinformatics* **2005**, *21*, 3787–3793.

21. Mistry, J.; Chuguransky, S.; Williams, L.; Qureshi, M.; Salazar, G. A.; Sonnhammer, E. L. L.; Tosatto, S. C. E.; Paladin, L.; Raj, S.; Richardson, L. J.; et al. Pfam: The protein families database in 2021. *Nucleic Acids Res.* **2021**, *49*, D412–D419.

22. Madeira, F.; Park, Y. M.; Lee, J.; Buso, N.; Gur, T.; Madhusoodanan, N.; Basutkar, P.; Tivey, A. R. N.; Potter, S. C.; Finn, R. D.; et al. The EMBL-EBI search and sequence analysis tools APIs in 2019. *Nucleic Acids Res.* **2019**, *47*, W636–W641.

23. Aramaki, T.; Blanc-Mathieu, R.; Endo, H.; Ohkubo, K.; Kanehisa, M.; Goto, S.; Ogata, H. KofamKOALA: KEGG Ortholog assignment based on profile HMM and adaptive score threshold. *Bioinformatics* **2020**, *36*, 2251–2252.

24. Tenenbaum, D. KEGGREST: Client-side REST access to KEGG. R package version 1.28.0. 2020.

25. Mihara, T.; Nishimura, Y.; Shimizu, Y.; Nishiyama, H.; Yoshikawa, G.; Uehara, H.; Hingamp, P.; Goto, S.; Ogata, H. Linking virus genomes with host taxonomy. *Viruses* **2016**, *8*, 66.

26. Petrushin, I.; Belikov, S.; Chernogor, L. Cooperative interaction of janthinobacterium sp. Slb01 and flavobacterium sp. slb02 in the diseased sponge lubomirskia baicalensis. *Int. J. Mol. Sci.* **2020**, *21*, 8123.

27. Payne, L. J.; Todeschini, T. C.; Wu, Y.; Perry, B. J.; Ronson, C. W.; Fineran, P. C.; Nobrega, F. L.; Jackson, S. A. Identification and classification of antiviral defence systems in bacteria and archaea with PADLOC reveals new system types. *Nucleic Acids Res.* **2021**, *49*, 10868–10878.

28. Couvin, D.; Bernheim, A.; Toffano-Nioche, C.; Touchon, M.; Michalik, J.; Néron, B.; Rocha, E. P. C.; Vergnaud, G.; Gautheret, D.; Pourcel, C. CRISPRCasFinder, an update of CRISRFinder, includes a portable version, enhanced performance and integrates search for Cas proteins. *Nucleic Acids Res.* **2018**, *46*, W246–W251.

29. Edwards, R. A.; McNair, K.; Faust, K.; Raes, J.; Dutilh, B. E. Computational approaches to predict bacteriophage-host relationships. *FEMS Microbiol. Rev.* **2016**, *40*, 258–272.
